# Supplementary material for: Rapid growth in Late Cretaceous sea turtles reveals life history strategies similar to extant leatherbacks
Source: PeerJ. 2023 Feb 10;11:e14864. doi: 10.7717/peerj.14864 (PMC9924133; doi:10.7717/peerj.14864)
Supplement: Supplemental Information 1 [file peerj-11-14864-s001.docx]

Supplementary Materials for

Title: Rapid growth in Cretaceous sea turtles reveals life history strategies similar to leatherbacks

Author: Laura E. Wilson

Correspondence to: [lewilson6@fhsu.edu](mailto:lewilson6@fhsu.edu)

**This PDF file includes:**

Supplementary Text

References

Figs. S1 to S4

Supplementary Text: Histologic descriptions

FHSM VP-17979, *Protostega gigas* humerus and femur (Fig. 2A-D; Fig. S1A-F)

Although bone shape and size vary between bones, similar histologic patterns are observed in both the femur and humerus of FHSM VP-17979. Bones lack a distinct medullary cavity with well-vascularized, spongiosa bone continuing through the entire section. Longitudinally-oriented primary osteons dominate both the femur and humerus, with some canals stretched radially or circumferentially in different parts of the section. Vascular canals are surrounded by lamellar- and parallel-fibered tissue with some woven tissue between osteons, producing a fibrolamellar complex (*sensu* Ricqles et al., 1991). Proportionately more bone is observed in the femur comparted to the humerus, likely related to lower absolute bone appositional rates due to smaller bone size and less functional pressure for rapid bone growth. The vasculature and spongiose nature of *Protostega*long bones are similar to the leatherback sea turtle (Rhodin, 1985; de Ricqlès, Castanet & Francillon‐Vieillot, 2004; Kriloff et al., 2008; Houssaye, Sander & Klein, 2016) (Figs. 3C, S4).  No secondary remodeling is observed.

Four cyclical growth marks (CGMs) are observed in both the femur and the humerus, with the fourth inconsistently visible along portions of the periosteal margin of the femur. Taphonomic alteration (crushing, microbial invasion, etc.) prevent clear analysis of the periosteal-most surface in sectioned specimens. The first and second GCMs of the humerus are visually similar to the depositional cycles Curry (1999) observed in the early juvenile *Apatosaurus* scapula. These growth marks lack a clear line delineating a full cessation of growth, as seen in lines of arrested growth (LAGs). CGMs marked by changes in deposition without a clear LAG are also typical of extant sea turtles early in ontogeny, like the Kemp’s ridley sea turtle (Snover & Hohn, 2004). Skeletochronology studies on known-aged sea turtles indicate that these marks should be considered annual an can be used to age individuals (Snover & Hohn, 2004). Similarities in marks and relative distance between CGMs in the humerus and femur also support that they represent annual cycles. Although there is some internal crushing, there is no evidence to suggest that there are missing CGMs due to taphonomy or resorption. Based on histological evidence, FHSM VP-17979 was in its fifth year of growth at the time of death.

CM 1393, *Protostega gigas* humerus (Fig. S1G-I)

CM-1393 has a significant amount of crushing that prevents analysis of a complete cross section. The best-preserved areas show a similar pattern to FHSM VP-17979 with large vascular spaces extending to the periosteal surface. The effects of crushing are likely amplified by the cancellous nature of the bone. Longitudinal primary osteons are surrounded by parallel-fibered and lamellar tissue with some woven bone between osteons, and they are arranged in concentric layers. No secondary remodeling is observed.

Despite crushing, two CGMs can be identified near the periosteal margin of the bone. CM 1393 was at least in its third year of growth at the time of death. Though likely (considering just the periosteal0most bone is clearly preserved), it is unknown if additional CGMs were present in the inner cortex prior to crushing. Overall, this age estimate is consistent with what is expected considering similarities in size between FHSM VP-17979 and CM 1393 (Fig. 1; Table 1) , especially if additional GCMs were destroyed during post-mortem crushing.

CM 1421, *Protostega gigas* humerus (Fig. S2A-C)

Despite being almost twice as large as FHSM VP 17979 and CM 1393 (Table 1), the bone microstructure patterns observed in CM 1421 are strikingly similar. CM 1421 is comprised of spongiosa bone that extends to the periosteal surface with no clearly delineation medullary cavity. Vascular patterns are similar to those observed in smaller *Protostega* specimens, with large longitudinally-oriented primary osteons arranged in concentric layers; vascular canals increase in organization towards the periosteal surface. Likewise, fibrolamellar bone with a mix of woven bone between abundant primary osteons is observed. Minimal secondary remodeling is evident by the formation of large erosion rooms throughout the bone cross section.

Despite crushing and bacterial invasion that obscures some areas of the inner-most bone, five CGMs are identified in the outer bone tissue extending to the periosteal margin. Retrocalculation performed by scaling and superimposing the smaller FHSM VP-17979 to the larger CM 1421 indicates that multiple CGMs were lost due to secondary remodeling and taphonomic alteration of CM 1421. Although estimates could be skewed due to the variable nature of CGM deposition in sea turtles (illustrated by the inconsistency in bone apposition rates between CGMs of FHSM VP-17979), it is estimated that three CGMs could have been lost. This indicates that CM 1421 was possibly in its ninth year of life at the time of death. No external fundamental system (EFS) was identified along the periosteal margin of the bone, indicating this individual was still actively growing at the time of death.

KUVP 1208, *Protostega gigas* humerus (Fig. 2E-F; Fig. S2D-F)

KUVP 1208 is the largest specimen in this study, though only slightly larger than CM 1421. Unsurprisingly given the similar sizes, the bone microstructures of KUVP 1208 and CM 1421 are also quite similar. Like all the humeri in this study, post-depositional crushing obscures the innermost bone tissue. However, KUVP 1208 does preserve spongiose bone throughout the humerus with the size and density of vascular canals decreasing towards the periosteal surface. Although bone is denser moving periosteally, it is still more spongiose than living hard shelled sea turtles, the more basal *Desmotochelys*, and *Toxochelys* (see below). Like other *Protostega* elements sampled in this study, vascularization is arranged circumferentially. Under polarized light, a mixture of parallel-fibered and woven collagen fiber orientation with a higher degree paralleled-fibered bone is observed throughout the cortex; parallel and lamellar bone is particularly concentrated around vascular canals.

Six CGMs can be identified in the humeral cross-section. However, retrocalculation using FHSM VP-17979 identifies at least two CGMs lost due to crushing and/or remodeling. This means the individual was at least in its night year of life at the time of death, similar to CM-1421. As in other specimens, CMGs are not evenly spaced. Also similar to CM 1421, KUVP 1208 is lacking an EFS and has vascular canals that open to the periosteal surface indicating KUVP 1208 was still growing at the time of death.

FHSM VP-17470, *Desmatochelys lowii* humerus (Fig. 3A; Fig. S3A-C)

*Desmatochelys* FHSM VP-17470 has a spongiosa medullary region that gradually transitions into denser cortical bone periosteally. The cortical region is well vascularized with longitudinal primary osteons surrounded by woven- and parallel-fibered tissue and arranged in concentric rows, forming fibrolamellar bone. Vascular canals are stretched circumferentially in some regions. The cortex of *Desmatochelys* does not have the spongiosa texture of *Protostega*, but has more compact cortical bone with smaller and fewer vascular canals. There is extensive secondary remodeling within the inner cortex and where the cortex transitions to the medullary region, but it is not clear if any or how many GCMs were removed by remodeling. Interestingly, secondary osteons seem to be replacing primary osteons using the original vascular canals without removing growth marks. This bone replacement pattern has also been observed in cetacean ribs (H. Woodward pers. comm. 2022). Fifteen CGMs are observed and are significantly more closely spaced than CGMs observed in *Protostega* specimens indicating slower bone apposition rates. Consequently, FHSM VP-17970 was at least in its 16^th^ year at the time of death. Vascularized bone tissue continues to the periosteal margin with no evidence of an EFS, indicating the individual was still growing at the time of death.

FHSM VP-700, *Toxochelys latiremis* humerus (Fig. 3B; Fig. S3D-F)

Bone microstructure patterns in *Toxochelys* FHSM VP-700 are more similar to *Desmatochelys* than *Protostega*. Like *Desmatochelys*, a spongiosa medullary area transitions into denser cortical bone towards the periosteal surface. Longitudinal and circumferential vascular canals are small but abundant and become more circumferentially arranged towards the periosteal surface. Some parallel-fibered and abundant woven bone are observed through the section. Large erosion rooms are present in the inner part of the bone and are often surrounded by lamellar bone. Although the number of CGMs preserved varies throughout the bone depending on the distribution of spongiosa bone and secondary remodeling, at least five CGMs are observed. However, it is unknown how many CGMs may be lacking due to endosteal resorption and secondary remodeling. An avascular layer with smaller osteocyte lacuna and parallel to lamellar bone does surround the periosteal surface of the humerus. This is possibly an EFS, indicating the FHSM VP-700 individual had reached somatic growth at the time of death.

**References**

Curry KA. 1999. Ontogenetic histology of *Apatosaurus* (Dinosauria: Sauropoda): New insights on growth rates and longevity. *Journal of Vertebrate Paleontology* 19:654–665.

Houssaye A, Sander MP, Klein N. 2016. Adaptive patterns in aquatic amniote bone microanatomy—more complex than previously thought. *Integrative and Comparative Biology* 56:1349–1369. DOI: 10.1093/icb/icw120.

Kriloff A, Germain D, Canoville A, Vincent P, Sache M, Laurin M. 2008. Evolution of bone microanatomy of the tetrapod tibia and its use in palaeobiological inference. *Journal of Evolutionary Biology* 21:807–826. DOI: 10.1111/j.1420-9101.2008.01512.x.

Rhodin AGJ. 1985. Comparative chondro-osseous development and growth of marine turtles. *Copeia* 1985:752–771. DOI: 10.2307/1444768.

de Ricqlès A, Castanet J, Francillon‐Vieillot H. 2004. The ‘message’ of bone tissue in paleoherpetology. *Italian Journal of Zoology* 71:3–12. DOI: 10.1080/11250000409356599.

Ricqles A de, Meunier FJ, Castanet J, Francillon-Viellot H. 1991. Comparative microstructure of bone. In: Hall BK ed. *Bone*. Boca Raton: CRC Press, 1–78.

Snover ML, Hohn AA. 2004. Validation and interpretation of annual skeletal marks in loggerhead (*Caretta caretta*) and Kemp’s ridley (*Lepidochelys kempii*) sea turtles. *Fishery Bulletin* 102:682–693.


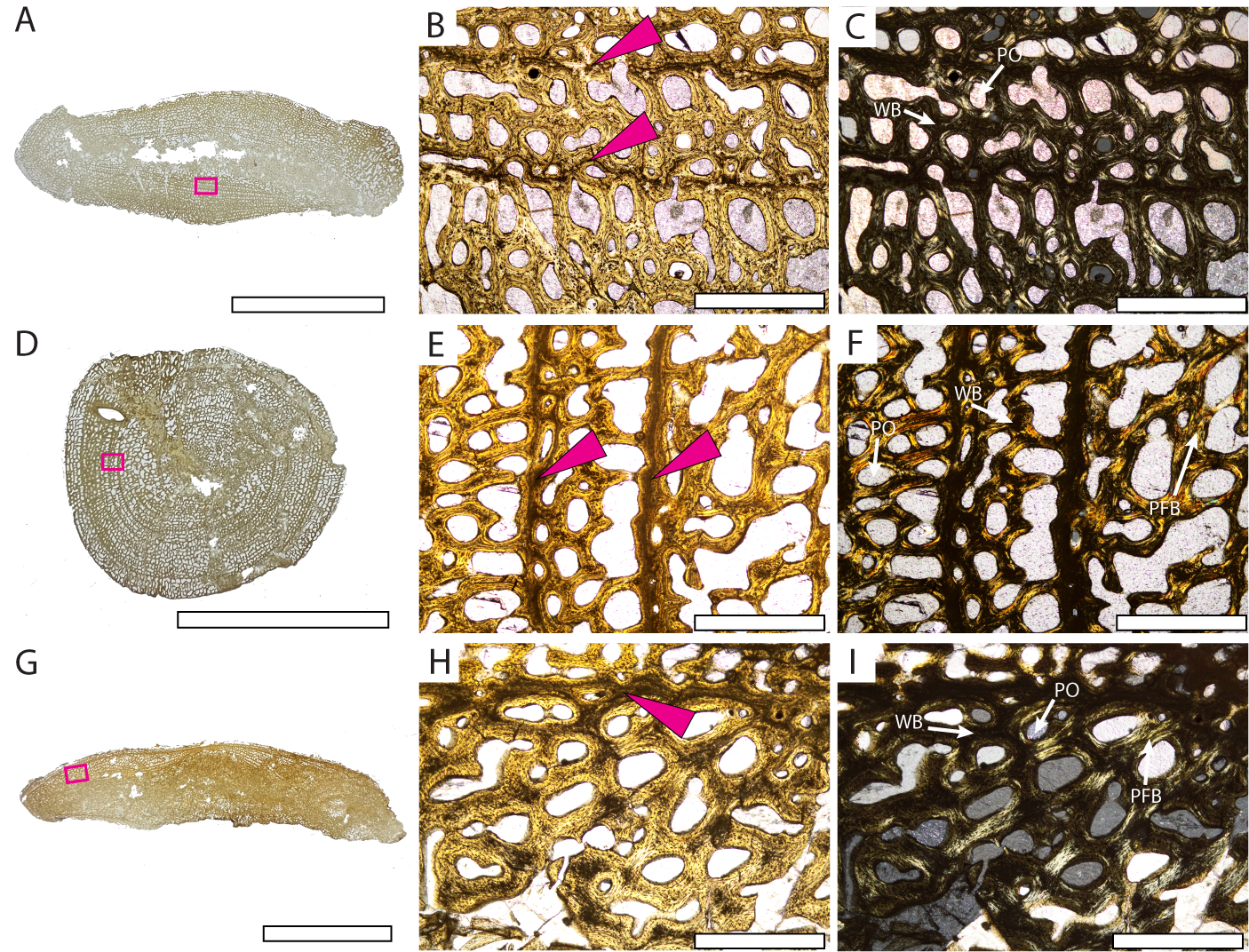


Fig. S1. Long bone microstructure of small *Protostega* specimens. *P. gigas* FHSM VP-17979 humerus full section in plane light (A), and close-up of bone microstructure in plane (B) and polarized light (C). *P. gigas* FHSM VP-17979 femur full section in plane light (D), and close-up of bone microstructure in plane (E) and polarized light (F). *P. gigas* CM 1393 humerus full section in plane light (G), and close-up of bone microstructure in plane (H) and polarized light (I). Scale bar on A, D, and G is 2cm; scale bar on B, C, E, F, H and I is 1mm. Pink arrow: CGMs, PO: primary osteon, WB: woven bone, PFB: parallel-fibered bone. High-resolution images available on Morphobank Project 4289.


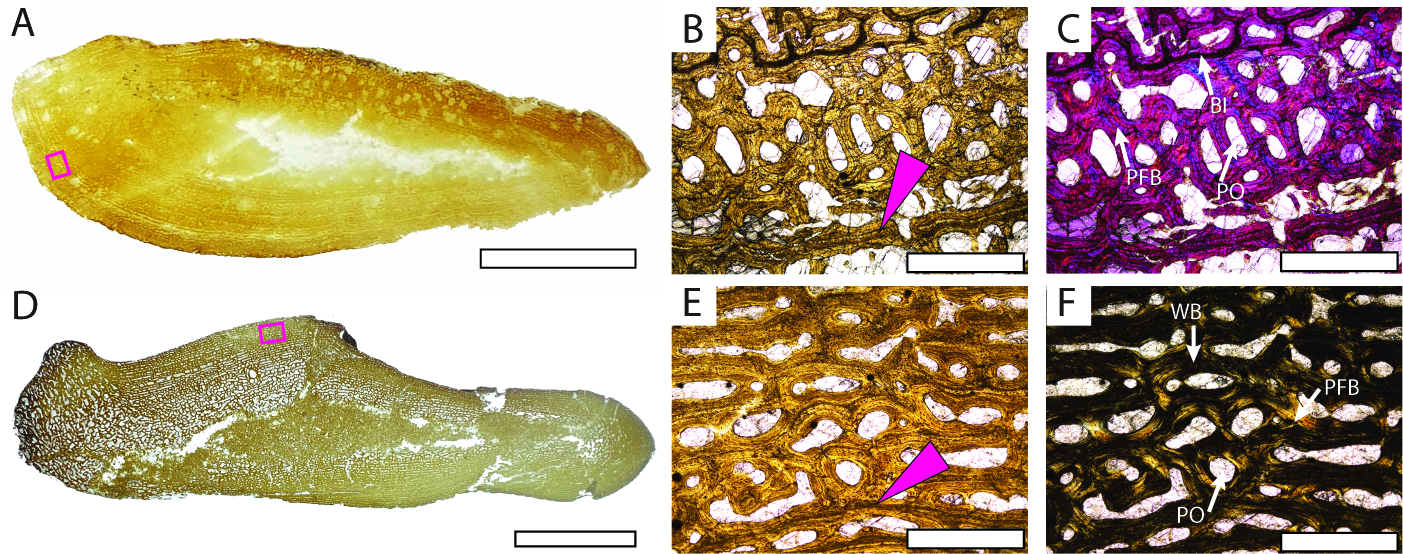


Fig. S2. Humerus microstructure of large *Protostega* specimens. *P. gigas* CM 1421 full section in plane light (A), and close-up of bone microstructure in plane (B) and polarized light with a lambda filter (C). *P. gigas* KUVP 1208 full section in plane light (D), and close-up of bone microstructure in plane (E) and polarized light (F). Scale bar on A and D is 2cm; scale bar on B, C, E, and F is 1mm. Pink arrow: CGMs, PO: primary osteon, WB: woven bone, PFB: parallel-fibered bone, BI: bacterial invasion. High-resolution images available on Morphobank Project 4289.


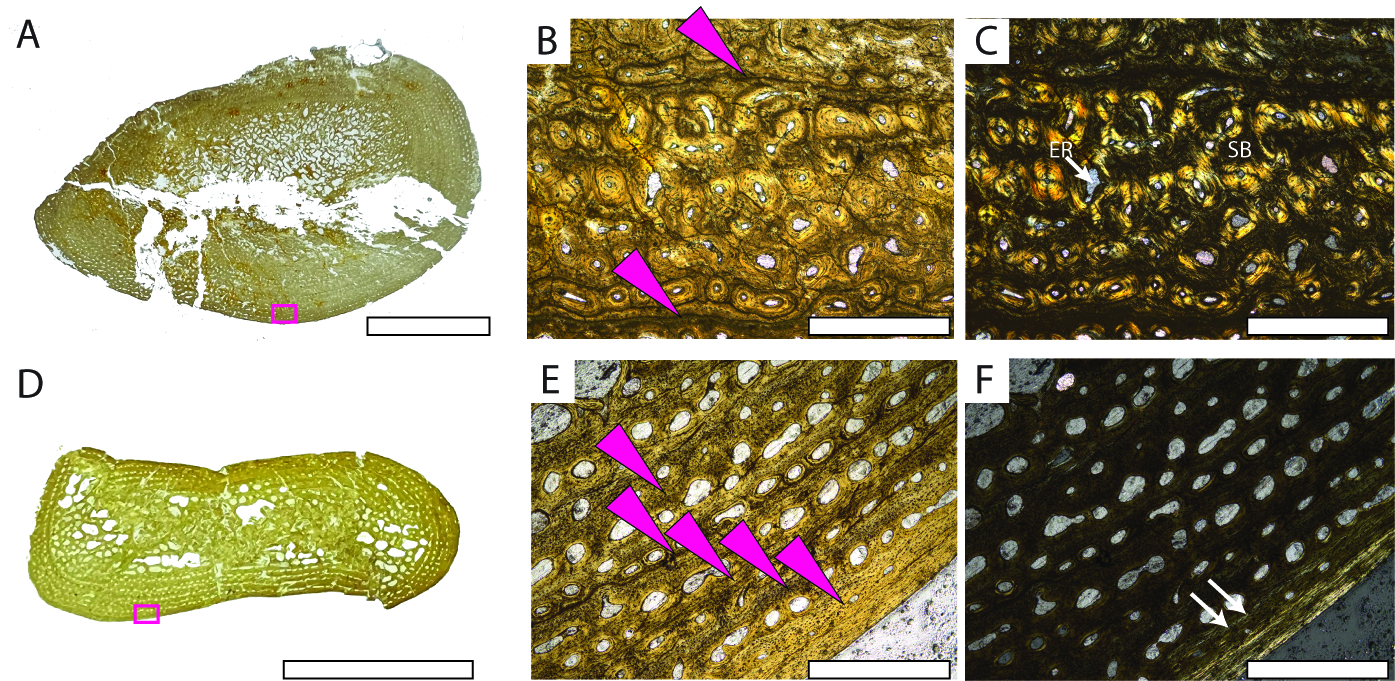


Fig. S3. Humerus microstructure of non-Protostega sea turtles for comparison. Basal protostegid *Desmatochelys lowii* FHSM VP-17470 full section in plane light (A), and close-up of bone microstructure in plane (B) and polarized light (C). Non-protostegid basal sea turtle *Toxochelys latiremis* FHSM VP-700 full section in plane light (D), and close-up of bone microstructure in plane (E) and polarized light (F). Double white arrow marks a possible EFS along the periosteal surface of FHSM VP-700. Scale bar on A and D is 1cm; scale bar on B, C, E, and F is 1mm. Pink arrow: CGMs, ER: erosion room, SB: secondary bone. High-resolution images available on Morphobank Project 4289.

Fig. S4. Full section of *Dermochelys coriacea*, the modern leatherback sea turtle. Specimen CRF 4911 was a female with a curved carapace length of 135 cm at the time of death. Growth marks are indicated by black arrows. Scale bar is 2cm.­­
